# Differences in healthcare structures, processes and outcomes of neighbouring European countries: the example of Germany and the Netherlands

**DOI:** 10.1007/s43999-023-00031-9

**Published:** 2023-11-17

**Authors:** Lars Schwettmann, Axel Hamprecht, Gesine H. Seeber, Stefan Pichler, Andreas Voss, Lena Ansmann, Falk Hoffmann

**Affiliations:** 1 Division Health Economics, Department of Health Services Research, School of Medicine and Health Sciences, Carl von Ossietzky University Oldenburg, Oldenburg, 26111 Germany; 2 Cross-Border Institute of Healthcare Systems and Prevention (CBI), University of Oldenburg, Oldenburg, Germany; 3 Institute of Medical Microbiology and Virology, School of Medicine and Health Sciences, Carl von Ossietzky University Oldenburg, Oldenburg, Germany; 4 University Hospital for Orthopaedics and Trauma Surgery Pius-Hospital, Medical Campus University of Oldenburg, Oldenburg, Germany; 5 Department of Orthopaedics, University of Groningen, University Medical Center Groningen, Groningen, Netherlands; 6 Cross-Border Institute of Healthcare Systems and Prevention (CBI), University of Groningen, Groningen, Netherlands; 7 Department of Economics, Econometrics and Finance, University of Groningen, Groningen, Netherlands; 8 Department of Medical Microbiology and Infection Prevention, University Medical Centre Groningen, Groningen, Netherlands; 9 Department of Health Services Research, School of Medicine and Health Sciences, Division Organisational Health Services Research, Carl von Ossietzky University Oldenburg, Oldenburg, Germany; 10 Chair of Medical Sociology, Institute of Medical Sociology, Health Services Research and Rehabilitation Science (IMVR), Faculty of Medicine, University of Cologne, Cologne, Germany; 11 Division Outpatient Care and Pharmacoepidemiology, Department of Health Services Research, School of Medicine and Health Sciences, Carl von Ossietzky University Oldenburg, Oldenburg, Germany

**Keywords:** Health systems, Cross-border, Delivery of health care, Germany, Netherlands

## Abstract

Although healthcare systems across Europe face rather similar challenges, their organization varies widely. Even neighbouring countries substantially differ with respect to healthcare structures, processes, and resulting outcomes. Focusing on Germany and the Netherlands as examples of such neighbouring countries, this paper will first identify and discuss similarities and major differences between both systems on the macro-level of healthcare. It further argues that it is often unknown how these differences trickle down to individual healthcare organizations, providers, patients or citizens, i.e., to the meso- and micro-level of healthcare. Hence, in a second step, potential implications of macro-level differences are described by considering the examples of total hip arthroplasty, antibiotic prescription practices and resistance, and nursing home care in Germany and the Netherlands. The paper concludes with an outlook on how these differences can be studied using the example of the project “Comparison of healthcare structures, processes and outcomes in the Northern German and Dutch cross-border region” (CHARE-GD). It further discusses potential prospects and challenges of corresponding cross-national research.

## Introduction

Many European countries face similar challenges related to their healthcare systems; these challenges continued to exist during and after the COVID-19 pandemic (according to the Organisation for Economic Cooperation and Development (OECD) [1]). On the one hand, the pandemic has drawn attention to the need for more resilient health systems. On the other hand, some long-term challenges were concealed in previous years and are now resurfacing with more urgency than before. This concerns especially the consequences of societal ageing, leading to a higher prevalence of age-related diseases, the need for new forms of care in different domains, including informal care, and constantly increasing financial burdens.

Although they face similar challenges, many European countries have developed rather different strategies to tackle them. For this reason, the structure and organization of healthcare varies widely across Europe. Researchers in the field of health system comparisons, foremost the European Observatory on Health Systems and Policies [2], comprehensively examined similarities and differences in healthcare systems between countries. They conclude that even neighbouring countries substantially differ concerning healthcare structures and processes, ultimately resulting in different outcomes for patients and the healthcare system.

Germany and the Netherlands are classic examples of such neighbouring countries. Both systems clearly rank high among high-income countries in terms of the central aspects of healthcare system performance, which include quality, choice and equity, science and technology, and fiscal sustainability [3, 4]. However, there are remarkable differences when it comes to healthcare processes and outcomes, with the Dutch healthcare system often performing better than its German counterpart [4]. Health system comparisons typically focus on national similarities and differences in healthcare by presenting information on the macro-level [2, 4].

**Fig. 1:**
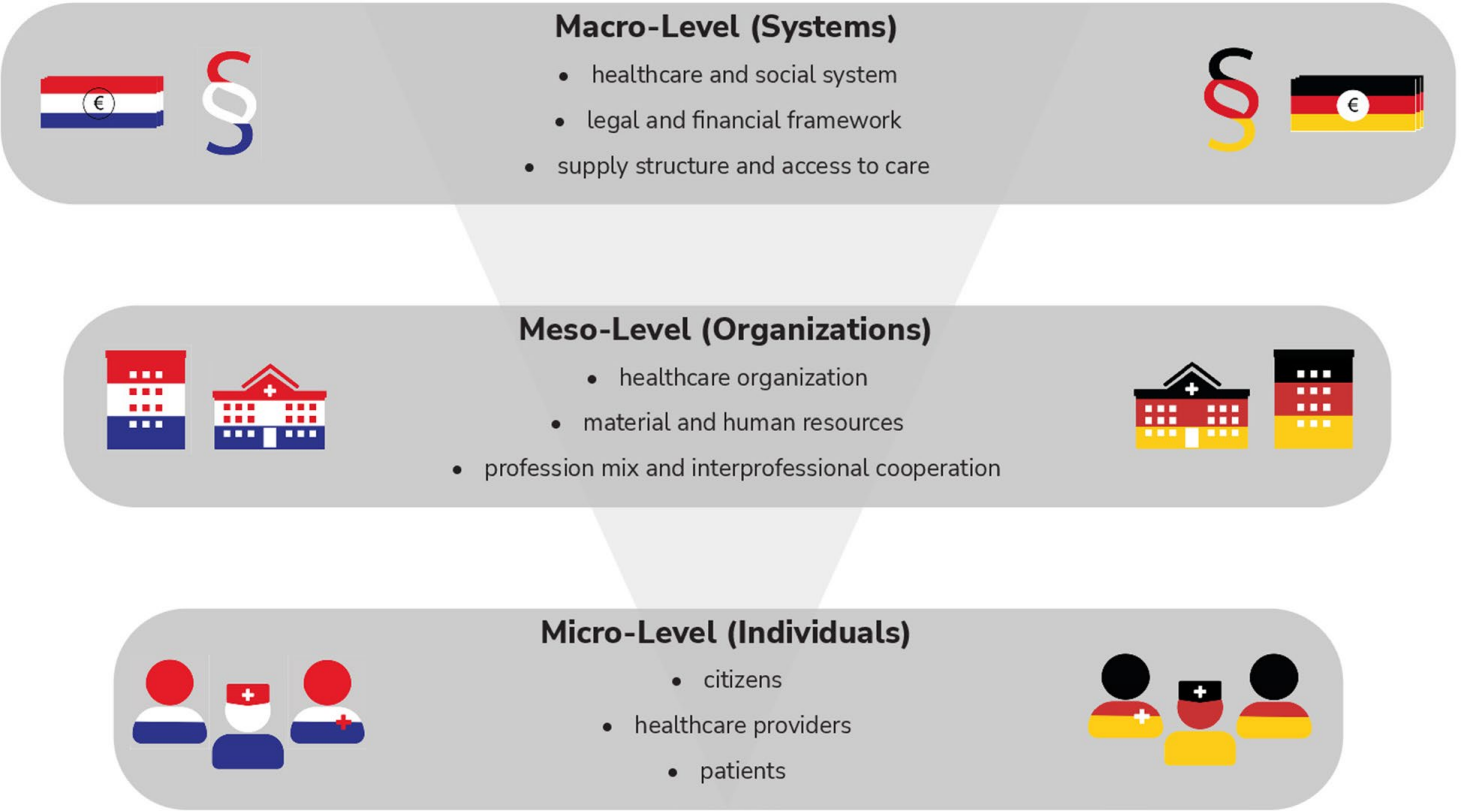
Description and examples of the three different levels of healthcare systems

Figure 1 provides a description and typical examples of the macro-, meso- and micro-level of healthcare. Data on the macro level concerns, for example, information on the organization of care, the financing of care, which also comprises decisions on the health insurance catalogue, and the delivery of care, including the role of the main actors or expenditures. Generally, standardized and robust macro-level data is relatively easy to access and compare. Particularly due to limited available data, it is more difficult to evaluate the implications of these differences on the meso-level in terms of specific healthcare organizations or on the micro-level with regard to providers, patients, and citizens. Consequently, much less is known about the corresponding similarities and differences between neighbouring countries at these levels.

The present article is a result of a longstanding cooperation of researchers from both sides of the Dutch-German border and reports experiences and findings from joint projects on differences in healthcare structures, processes and outcomes at the meso- and micro-level. First, it provides an overview of similarities and differences on the macro-level between the German and Dutch healthcare systems. Afterwards, it describes how the implications of these distinctions are reflected on the meso- and micro-level of healthcare. Furthermore, it gives an outlook on how such differences can be studied, discusses the potential of such cross-national research, and describes challenges researchers in corresponding projects may face.

### Similarities and differences in the organization of the German and Dutch health systems

When it comes to resources put into the health system, health expenditures in Germany are among the highest in the world. In 2020, they comprised 12.8% of the gross domestic product (GDP), while the corresponding value in the Netherlands was 11.1% [1]. Regarding life expectancy as a rough outcome measure, new-born boys and girls in the Netherlands in 2020 had a life expectancy of 79.7 and 83.1 years, respectively. In contrast, corresponding German values equalled 78.5 and 83.5 years. Interestingly, healthy life years, i.e., the expected number of years spent without any long-term activity limitation, are slightly higher for German new-borns (boys 64.7, girls 66.8 years) compared to Dutch new-borns (boys 62.4, girls 59.6 years). Hence, it is crucial to understand how health systems, as one potential determinant, are organized to use available resources.

The German and Dutch health systems are traditionally described as classic examples of the Bismarckian health system which have adopted some competitive elements [5, 6]. In both countries, a basic insurance benefits package exists, which is defined by the nongovernmental Federal Joint Committee for statutory health insurance funds (G-BA) in Germany and by the national government in the Netherlands. Both systems are characterized by competing non-profit mandatory universal sickness funds (Germany) or health insurances (Netherlands) with open enrolment and free choice of both insurers and providers. National health budgets have been established that are financed either by compulsory wage contributions of employers and employees, additional income-dependent contributions, and tax revenue (Germany) [7] or earmarked payroll tax from employers, community-rated premiums from individuals, tax revenue, and government grants (Netherlands) [8]. Additionally, the two systems apply risk-adjustment schemes among insurers, and hospitals are remunerated according to nationally-adapted diagnosis-related group (DRG) systems [5].

Nevertheless, remarkable differences in the organization of both systems exist. First and foremost, the German statutory health insurance (SHI) system is characterized by its corporatist mode of regulation. The government determines the general framework of reference, but the direct delivery of healthcare is organized by associations representing sickness funds or providers, which are all participating in the G-BA [7]. In the Netherlands, in contrast, the national government regulates health insurers and healthcare providers.

Furthermore, in Germany, individuals above a certain income level (viz 66,600 Euro per year in 2023) and civil servants can opt out of the SHI and choose supplementary fully private insurance without any governmental subsidies. In 2021, about 10.5% of the German population were members of such private health insurances.^1^ In the Netherlands, all residents have to purchase basic statutory insurance coverage from private health insurers. However, 84% of them also buy complementary coverage for relevant benefits not included in the basic statutory package, while corresponding schemes in Germany only include minor benefits [8]. Additionally, adults in the Netherlands have to pay an annual deductible on healthcare costs (with a minimum of 385 Euro in 2023) except for services from primary care physicians and preventive services, while in Germany some co-payments exist, e.g., for inpatient services, drugs, and dental care. This deductible can be further increased if patients are willing to take the associated risk and want to obtain a discount on their premiums.

Other differences concern health care delivery. Primary care physicians in the Netherlands act as gatekeepers, and their offices are privately owned [8, 9]. Patients need referrals to receive specialised treatment, which is usually offered by hospital-based specialists. Furthermore, hospitals are generally under non-governmental non-profit ownership. In 2020, there were on average 2.9 hospital beds per 1,000 inhabitants [1]. In Germany, in contrast, patients are free to choose among both primary physicians and outpatient specialist physicians, who usually work in private practices [9]. Hence, gatekeeping is only implemented in some freely chosen sickness fund schemes. Additionally, about two-thirds of German hospitals are public, 25% are private for-profit, and 10% are private non-profit. Interestingly, the average number of hospital beds per 1,000 inhabitants has reached 7.8 in Germany, thus indicating a further remarkable structural difference in healthcare provision [1]. To a certain extent, this difference can be explained by more outpatient and day treatments in the Netherlands which do not require an overnight stay.

As part of the social insurance system, German long-term care insurance is financed by income-dependent membership contributions. It covers only part of the actual costs incurred, while the rest is paid either by private co-payments or, in case of financial need, by social welfare [10]. The Dutch long-term care insurance is mandatory for all inhabitants and financed by the general income tax. When claiming benefits, there is also a nationwide uniform co-payment, the amount of which depends on sociodemographic factors and the care setting [11]. In 2019, total spending on long-term care in Germany was about 2.2% of GDP compared to 4.1% in the Netherlands [12]. In Germany, long-term care insurance pays for services only if a person depends on assistance in managing everyday tasks such as personal hygiene, nutrition, or mobility due to physical, mental, or emotional limitations. To receive benefits from long-term care insurance, this need for care must be assessed by an assessor from the Medical Service. Based on the assessment, a care grade (1–5) is then determined, which reflects the severity of the need for care [13]. In the Netherlands, the definition of the need for care is similar to that in Germany, and eligibility is also assessed by an independent central assessment agency, which assigns individuals to one of ten care packages [14]. About 4.1 million persons in Germany claimed long-term care insurance benefits in 2019. Of these, about 3.3 million received care at home (80.5%), while about 800,000 were in nursing homes (19.5%) [15]. In the Netherlands, about 278,000 citizens received long-term care benefits. Of these, 79,700 persons were cared for at home (28.7%), and 198,400 beneficiaries lived in care facilities (71.4%) [16].

Finally, the Dutch health system is specifically committed to providing a great level of information to decision-makers and patients [5]. For example, the National Healthcare Institute (ZiNL) regularly publishes various performance measures in reports and on a website to enable evidence-based policy decisions and strengthen patient empowerment. However, compared to the Netherlands, corresponding information in Germany is rather scarce and is utilized less by patients. One prominent exemption is the visibility of the Institute for Quality and Efficiency in Health Care (IQWiG), which informs both the Federal Joint Committee and patients about the benefits and harms of medical interventions.

In summary, although the German and the Dutch systems have belonged to the same family of health systems for a long time, substantial differences at the macro-level are visible. Yet, it is not clear how these differences affect the meso- and micro-level.

### Differences in care processes and outcomes

To describe differences in healthcare structures, processes, and outcomes on both sides of the border, examples from three main healthcare sectors are presented, i.e., from hospitals (total hip arthroplasty), primary care (antibiotic prescription practices and resistance), and nursing homes (nursing home care).

#### The case of total hip arthroplasty

One example of differences between Germany and the Netherlands is the care received after total hip arthroplasty (THA). This procedure is a highly successful management option for end-stage hip osteoarthritis [17] and is one of the most frequently performed orthopaedic surgeries in both countries. The operation itself is highly standardized, but patient aftercare and rehabilitation approaches differ considerably between Germany and the Netherlands [18]. For example, in the Netherlands, THA aftercare is provided by the orthopaedic surgeon at the hospital where the operation took place. In contrast, in Germany, in-patient and out-patient care are separated, so that patients are usually followed up by an orthopaedic surgeon in private practice who did not perform the operation at the hospital. Moreover, in the Netherlands, most patients undergo fast-track surgery and are discharged into their home environment within a few days following THA. In combination with the fact that Dutch basic health insurance packages – determined at the macro health policy-level – do not cover postoperative physical therapy, the drawback of this approach is that patients receive less support during THA rehabilitation. In contrast, in the German system, patients usually stay in the hospital for about 8–10 postoperative days before being transferred to a specialized rehabilitation centre for a period of three weeks, where all costs are covered by basic SHI [18]. Following these three weeks at the rehabilitation centre, the treating physician can further prescribe physical therapy if needed. The number of physical therapy sessions paid by the SHI is unlimited for six months following THA.

Previous research comparing the different approaches to THA rehabilitation in Germany versus the Netherlands found conflicting results regarding clinical effectiveness and patient satisfaction. While we found that German patients had better functional outcomes and were more satisfied than Dutch patients at three and six months following THA (own unpublished data), a comparable study showed no significant differences [19]. This indicates that more intense post-operative health care (i.e., more extended hospital stays, more physical therapy) does not inevitably lead to better patient-reported outcomes after surgery. Previous research suggests that treatment expectations and patients’ self-efficacy influence THA outcomes [20–23], and both factors might differ between Dutch and German patients. Moreover, an integrated model of patient expectations by Laferton et al. conceptualizes the association between patient expectations and outcomes [24]. This model is in line with empirical findings suggesting that preoperative expectations, in combination with patient and health system characteristics differing between Germany and the Netherlands, influence postoperative THA outcomes in terms of satisfaction, physical function, and health-related quality of life [25].

#### The case of antibiotic prescription practices and resistance

Resistance to antibiotics is an increasing problem worldwide and is associated with higher mortality and increased length of stay in hospitals. Between 2007 and 2015, the annual burden of infections with antibiotic-resistant bacteria more than doubled across countries of the European Union/European Economic Area (EU/EEA) [26]. There are important differences between Germany and the Netherlands in infection control measures and the organisation of clinical microbiology, which likely have an impact on antibiotic resistance.

The most prominent and frequently cited example of differences in infection control strategies is patient screening for methicillin-resistant *Staphylococcus aureus* (MRSA). The Netherlands introduced a “search and destroy” strategy already in the mid-1980s; this strategy has resulted in low MRSA rates compared to neighbouring countries which started similar measures much later. In 2021, the frequency of MRSA among invasive *S. aureus* isolates was 4.9% in Germany compared to 1.5% in the Netherlands.^2^ However, when analysing resistance in other organisms, rates are more similar – e.g., for *Klebsiella pneumoniae*, resistance to 3^rd^ generation cephalosporins is almost identical (10.4% in Germany vs. 10.1% in the Netherlands in 2021).

In addition to infection control, the access to and organisation of clinical microbiology diagnostics as well as antibiotic stewardship differ significantly. In the Netherlands, the proportion of larger hospitals is higher. They often have their own microbiology laboratory and trained staff on-site, which leads to easy access, shorter transport times, and facilitates clinical consultations. In Germany, most hospitals have outsourced their microbiology laboratory to other (often private) providers, resulting in longer turn-around time for clinical samples and more difficult access to consultations by clinical microbiologists, which hinders the initiation of effective antimicrobial treatment and stewardship programmes.

Antibiotic consumption, however, is similar on the national level in Germany and the Netherlands – both countries are among those with the lowest consumption of antibiotics in the EU. In 2021, the community consumption of antibiotics was 7.6 defined daily doses per 1,000 inhabitants per day (DDD) in the Netherlands compared to 8.1 DDD in Germany (EU 15.0 DDD) [27]. Although these average figures in the outpatient sector are quite comparable, differences might be found within specific populations, settings or regions. For instance, a study on antibiotic prescription practices for children and adolescents in the northern Dutch-German border region showed a higher prescription prevalence in Germany compared to the Netherlands [28]. In addition, it has been shown that antibiotic prescription practices differ in border regions when compared to national averages [29].

#### The case of nursing home care

Several structural differences between both health systems affect nursing homes. As outpatient care in Germany is provided by primary care physicians and specialists solely working in private practice outside hospitals, both physician groups also offer home visits to households or nursing homes. Therefore, primary care physicians often continue to care for a patient after admission to a nursing home. In the Netherlands, specially trained elderly care physicians are employed by nursing homes, with one full-time nursing home physician solely caring for about 100 residents [30, 31]. Contrarily, on average, German primary care physicians care for 47 residents in 4 different nursing homes, in addition to working in their own practice [32].

In the Netherlands, psychologists, physiotherapists, speech therapists, and occupational therapists are all part of the nursing home team [30]. In Germany, in contrast, therapists work in private practice and offer home visits to nursing homes based on a physician’s prescription [33], which makes coordination of care more challenging. Furthermore, palliative care is well established in Dutch nursing homes, and end-of-life hospitalizations are rare [34]. Overall, a recent systematic review found large variations between countries worldwide in the proportion of nursing home residents who died in hospitals [35]. A closer look at recent studies even shows that only about 6% of Dutch [36], but almost 30% of German nursing home residents died in hospital [37]. These strikingly higher proportions for Germany were consistently found in all available studies, including during other periods of a nursing home stay [38]. Nevertheless, the organizational processes responsible for such differences and the consequences for both residents and healthcare providers remain unclear.

### A project for assessing these differences on the meso- and micro-level

As shown by our three examples, where differences between the structure or performance of the German and the Dutch health system are described, it is often unknown how these macro-level differences trickle down to individual healthcare organizations, providers, patients, or citizens. Therefore, the project “Comparison of healthcare structures, processes and outcomes in the Northern German and Dutch cross-border region” (CHARE-GD) aims to collect data on the meso-level of healthcare organizations as well as on the micro-level of providers, patients, and citizens. The project is funded by the Ministry of Science and Culture of Lower Saxony, including a total of ten different sub-projects, with a first funding phase (CHARE-GD I) extending from 2021 to 2024 and the second (CHARE-GD II) from 2022 to 2025. The general aim is to identify and explain observed differences between these two neighbouring European countries in different healthcare sectors/domains (e.g., primary care, hospital care, rehabilitation, and long-term care). Furthermore, a basis for an infrastructure will be developed to allow conducting reliable scientific comparisons of health and healthcare data.

The consortium includes interdisciplinary research teams from data science, social, clinical, public health, health economics, and health services research, mainly from the universities of Oldenburg (Germany) and Groningen (Netherlands). The cities of Oldenburg and Groningen are in the northern part of Western Europe, and they are located about 140 km apart. Rural areas divided by the border surround both cities. The corresponding cross-border region, the so-called Ems-Dollart region between the northeast of the Netherlands and the northwest of Germany, is home to about four million inhabitants and faces very similar societal challenges. Therefore, the Ems-Dollart region, as the northern cross-border region between Germany and the Netherlands, can be regarded as an excellent setting to study in detail how health system differences trickle down to healthcare providers, patients, and citizens. On the one hand, from a national point of view, such rural border regions often display specific challenges regarding health services provision since care utilisation as well as supply might be hampered by the border. On the other hand, the cooperation between researchers and healthcare providers from both sides of the border offers an excellent opportunity to consider aspects of cross-border healthcare.

### Potential and challenges of cross-border research

By using the northern Dutch-German cross-border region as a “living lab”, scientific evidence for improving healthcare across the border can be generated to inform healthcare improvement in cross-border regions. As a result, best practices and solutions on the meso- and micro-level may be transferred or developed in a bottom-up approach by starting on a regional level.

Based on previous projects and the first results from CHARE-GD, several challenges of cross-border research have already become apparent. One major challenge is that it is unclear whether differences between countries are “real” or due to differences in data collection methodologies. For instance, it is known that compared to the Netherlands, Germany has many more intensive care beds per inhabitant (33 vs. 6 intensive care beds per 100,000 inhabitants).^3^ However, this assumption is questioned because no unique definition of an “intensive care bed” exists. Consequently, many intermediate or intensive care beds in Germany might not be classified as such in the Netherlands.

The fact that available data might not be directly comparable can be a matter of concern in many projects. For example, in an earlier study investigating outpatient antibiotic prescription practices for children and adolescents in the cross-border region, two different data sources with their distinct limitations were available [28]. For Germany, data from one statutory health insurance company were used, but more than 150 statutory as well as private health insurance funds existed in Germany at the time the study was conducted. These funds differ regarding sociodemographic characteristics because several specific person groups are overrepresented in some of these funds [39, 40]. Such differences influence health services use and have already been found in children [41]. For the Netherlands, a pharmacy research database was used which includes information on all dispensed medications from about 55 community pharmacies, where citizens have to be registered. When compared with the source population recruited by a health insurance fund, which has more or less equal coverage across all regions, these pharmacies clearly represent different source populations, showing a high coverage of inhabitants around the pharmacy but relatively lower coverage in more distant regions.

Many more examples exist. For instance, no Dutch counterpart is available for the nationwide DRG (diagnosis-related groups) statistics, which provide data on all inpatients on an individual case level and are made available by the Research Data Centre of the Federal Statistical Office [42]. Another example concerns registry data. For instance, the Dutch national registry for orthopedic interventions (Landelijke Registratie Orthopedische Interventies; LROI) is mandatory and also collects patients’ self-reported outcome data on joint function, pain, or satisfaction. The German endoprosthetics registry (Deutsches Endoprothesenregister; EPRD), in contrast, is voluntary and does not collect subjectively reported information.

Another challenge arises from the fact that in the German system, follow-up data collected as part of routine medical care after surgery (e.g., THA) are stored both at the hospital providing care and at the private practice of the orthopaedic surgeon and/or general practitioner providing further treatment. In the Netherlands, in contrast, all case-associated data are stored at the treating hospital. This hampers data transfer between both countries since several different stakeholders are to be involved on the German side; their electronic health records are often unavailable, and even if they are, data linkage would lead to greater challenges with regard to data protection aspects.

### Outlook

On the example of the neighbouring European countries Germany and the Netherlands, we have shown how important it is to move from a health system to a health services research perspective and to ask how potential macro-level differences translate to the meso- and micro-level. The examples and challenges discussed here are relevant for any research on these aspects involving more than one country. Thus, in-depth research on these differences as well as efforts towards better comparability of data are needed. Keeping this in mind is essential as some of the differences on the macro-level also affect the possibility of collecting comparable data or recruiting via comparable methods. One must consider that setting up legal, ethical, and administrative frameworks for research and providing data infrastructures and access is even more challenging when different countries are involved. Approaching these issues for every single research question is time-consuming and makes it more difficult to answer the question of interest itself. Thus, emphasis should be placed on establishing comparable data infrastructures (e.g., prospective cross-national cohort studies, routinely collected or register data using the same protocols) to allow studying a broader range of research questions now and in the future. Finally, funding is needed for these kinds of projects; finding such funding can be challenging because funding organizations often solely support researchers in one country.

## Data Availability

Not applicable.

## Footnotes

1 https://www.vdek.com/presse/daten/b_versicherte.html (date last accessed: 09.03.2023).

2 http://atlas.ecdc.europa.eu/public/index.aspx (date last accessed: 30.03.2023).

3 https://www.destatis.de/DE/Themen/Gesellschaft-Umwelt/Gesundheit/Krankenhaeuser/Tabellen/eckzahlen-krankenhaeuser.html and https://www.venvn.nl/afdelingen/intensive-care/nieuws/ic-capaciteit-najaar-winter-2022-2023/ (date last accessed: 14.02.2023).
